# Supplementary material for: Design, Synthesis, In Silico and POM Studies for the Identification of the Pharmacophore Sites of Benzylidene Derivatives
Source: Molecules. 2023 Mar 13;28(6):2613. doi: 10.3390/molecules28062613 (PMC10053039; doi:10.3390/molecules28062613)
Supplement: Supplementary file 1 [file molecules-28-02613-s001.zip › molecules-2259658-supplementary.pdf]

Supplementary Materials

Design, Synthesis, Molecular Docking, Dynamics and POM Studies for the Identification of the Pharmacophore Sites of Benzylidene Derivatives

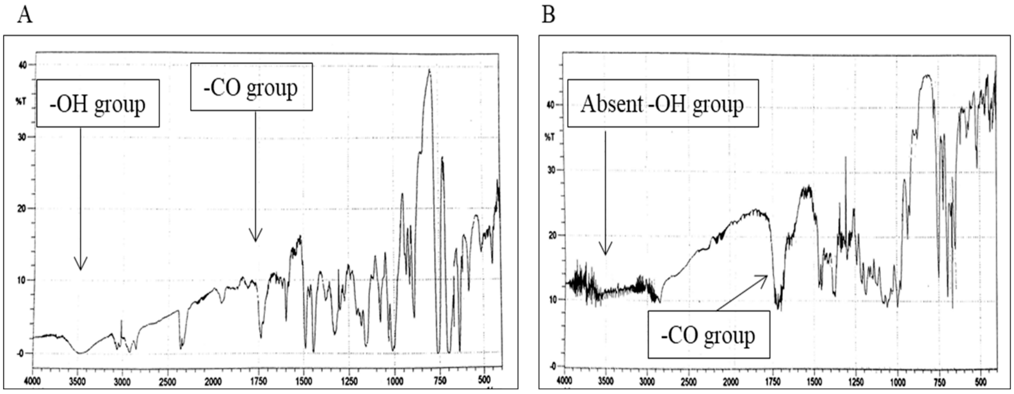

Figure S1. FTIR spectra of A: compound **3** and B: compound **4**.

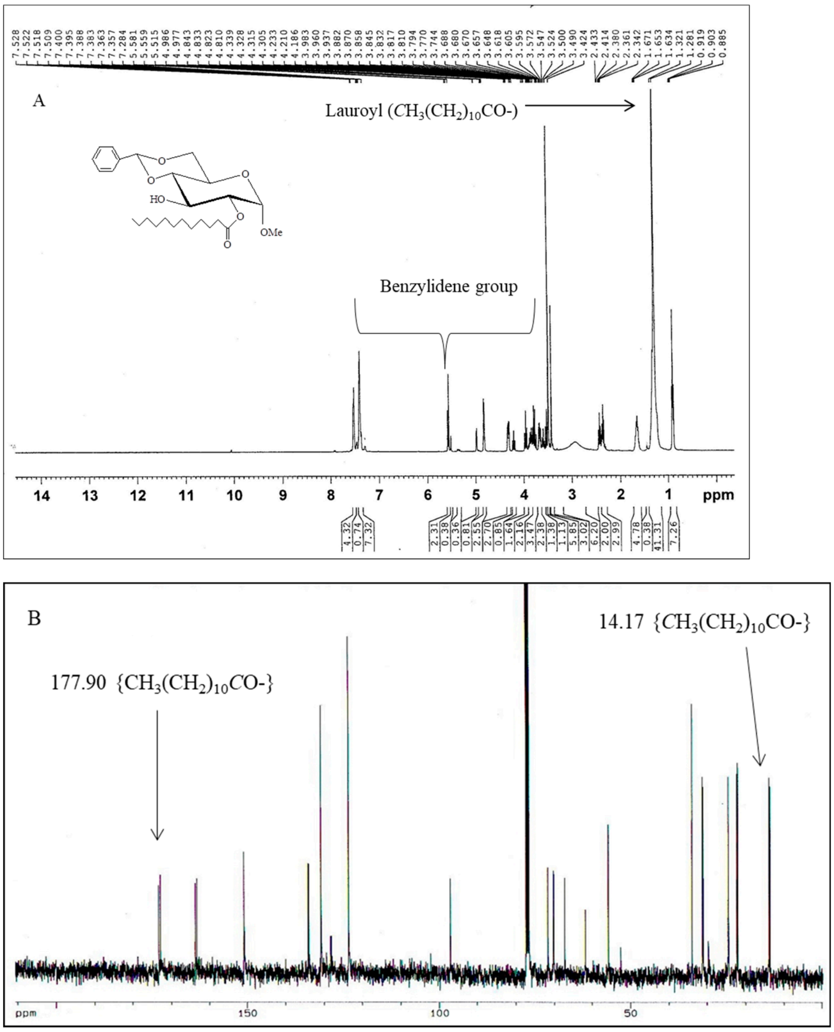

Figure S2. (**A**) ^{1}H-NMR and (**B**) ^{13}C-NMR spectra of the compound **3**.

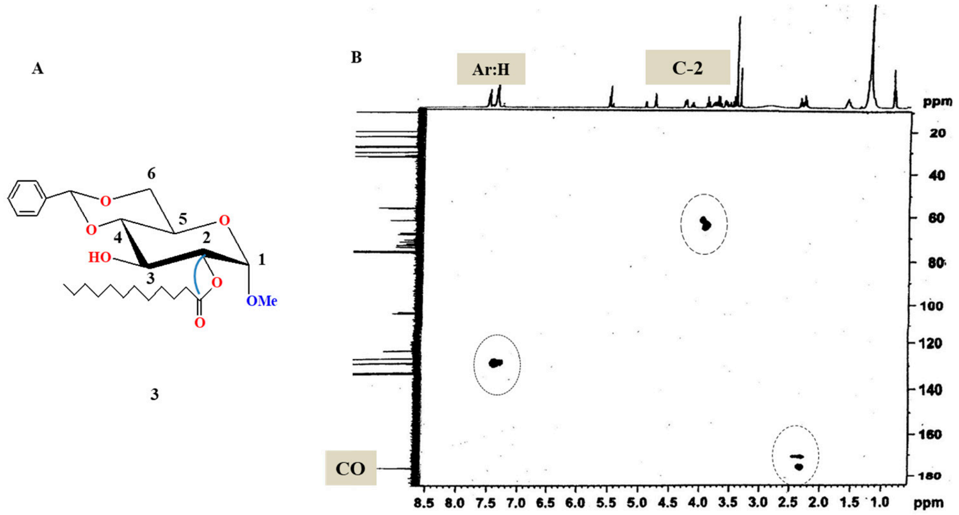

Figure S3. The HMBC correlations of (**A**) comppound **2** and (**B**) CO with H-2 and ArH protons.

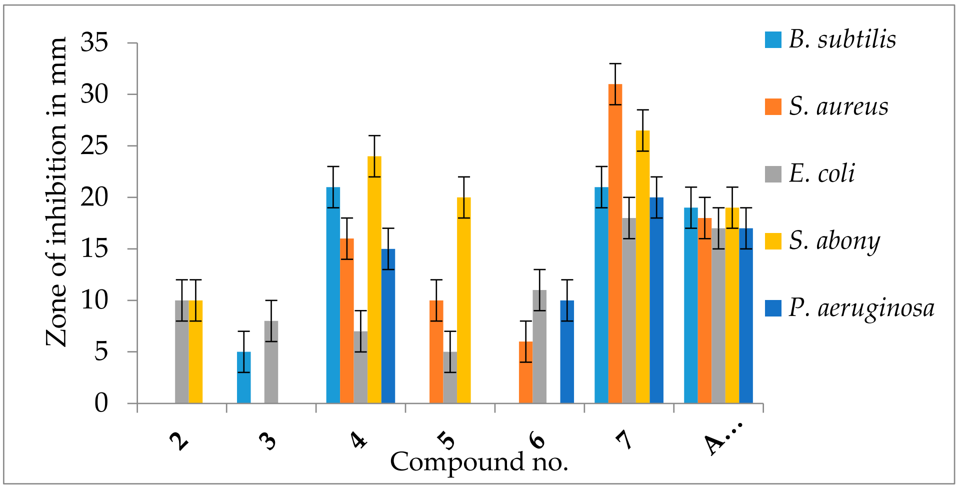

Figure S4. Zone of inhibition observed against both the Gram-positive and Gram-negative bacteria by the tested compounds.

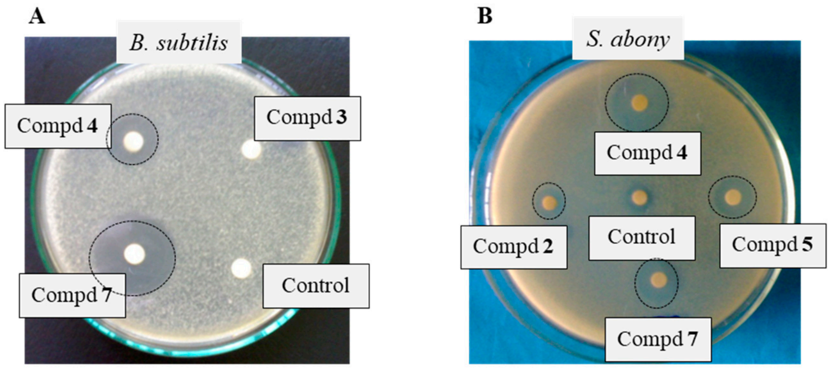

Figure S5. Inhibition zones were observed against **A**) *B. subtilis* by compounds **3**, **4**, and **7**; **B**) *S. abony* by compounds **2**, **4**, **5**, and **7**. Control (in DMSO) was treated as a negative control.

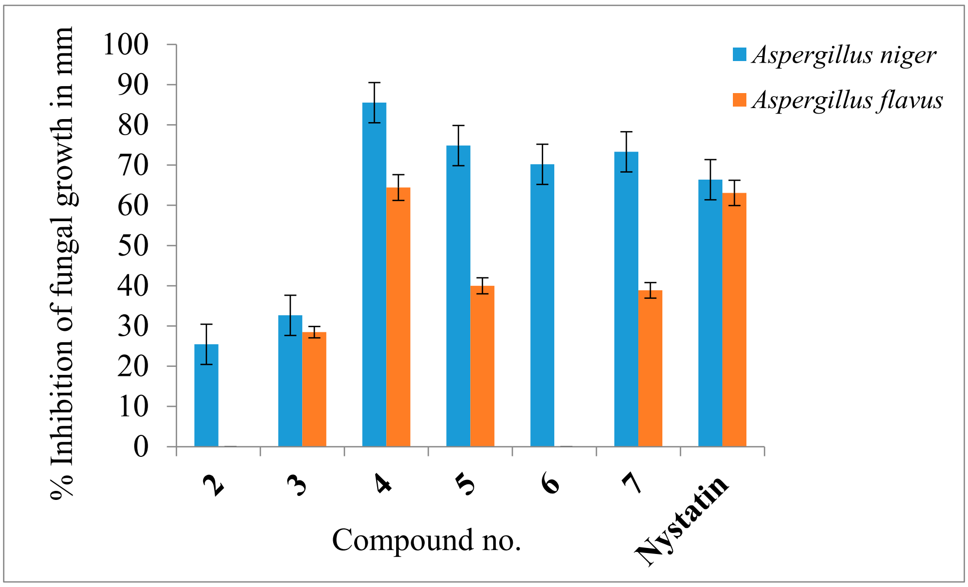

Figure S6. Antifungal activities of the synthesized test compounds.

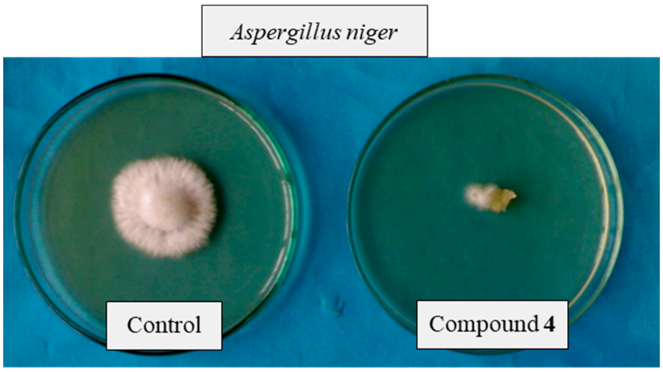

Figure S7. Inhibition of fungal growth observed in *A. niger* by the compound **4**.

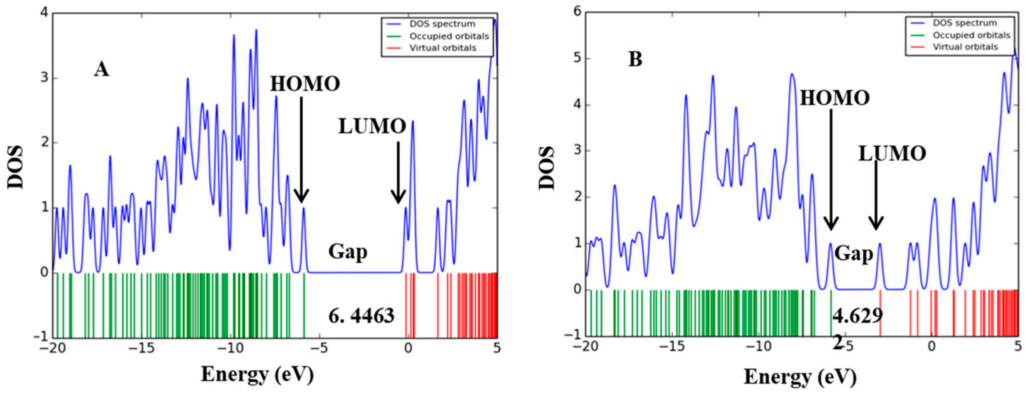

Figure S8. (**A**); DOS plot of compound **3** (**B**); DOS plot of compound **7**.

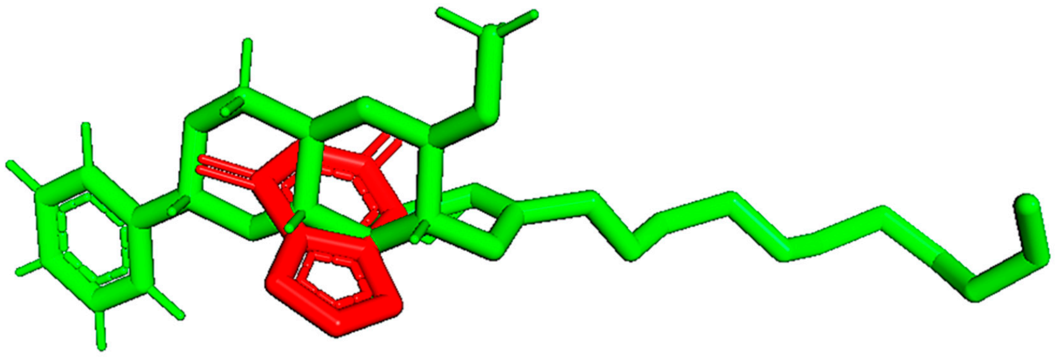

Figure S9. Re-docking pose with the RMSD value of < 2Å (Red = Original, Green = Docked).

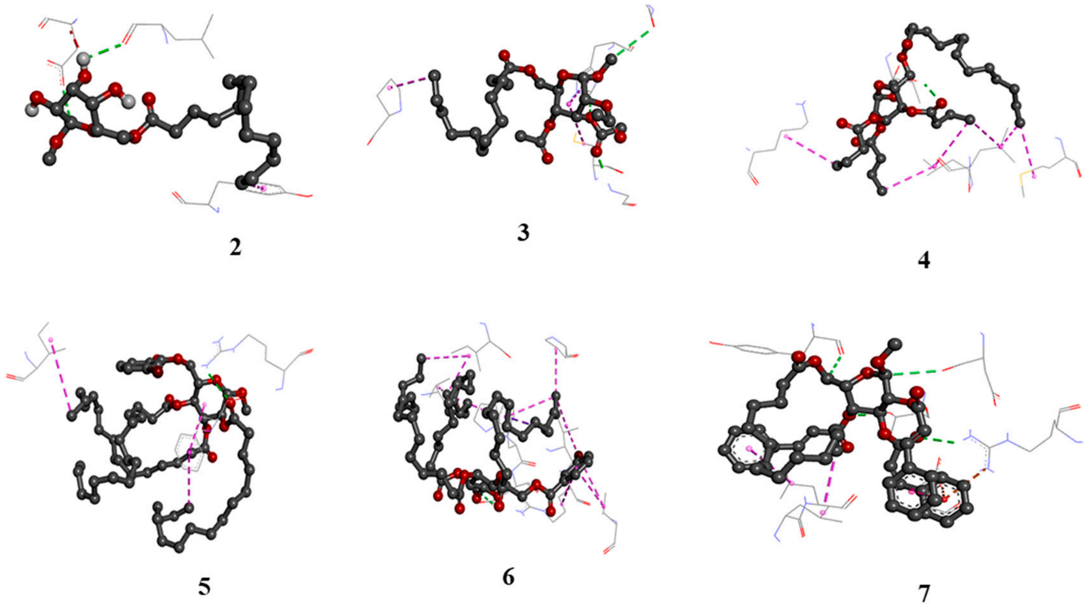

Figure S10. Nonbonding interactions of MGB derivatives (2-7) with 1KS5 generated by Discovery Studio.

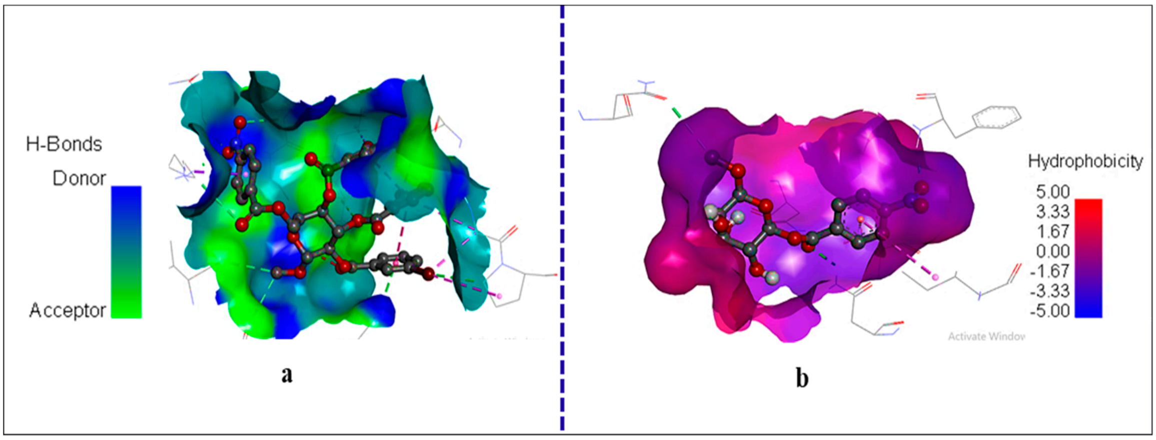

Figure S11. (**a**); Hydrogen bond surface of compound **7** (**b**); Hydrophobic bond surface of compound **7**.

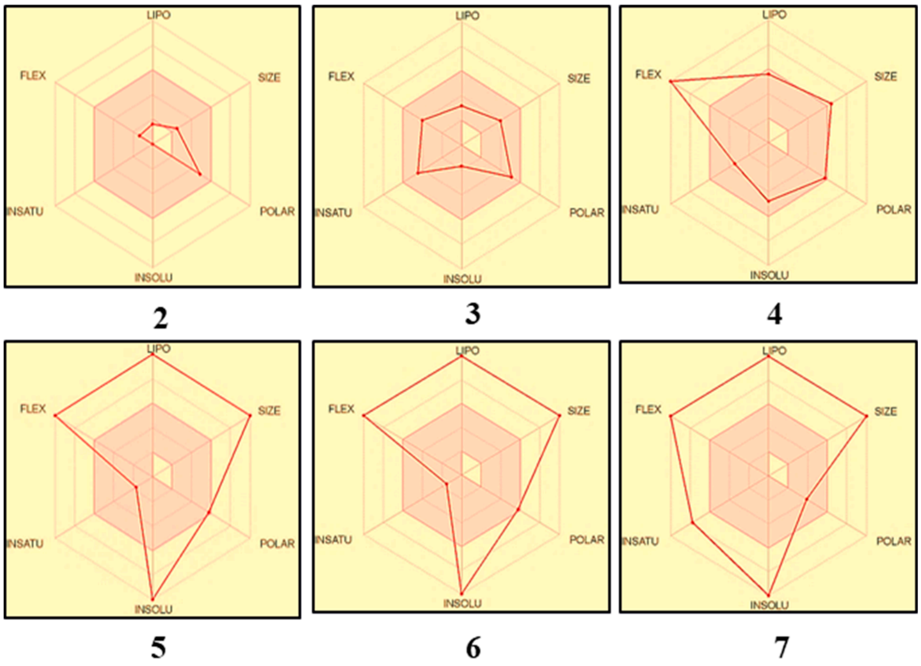

Figure S12. Bioactivity radar charts of the MGB derivatives (**2**-**7**) where FLEX: Flexibility, LIPO: Lipophilicity, INSATU: Insaturation and INSOLU: Insolubility.

Table S1 ^{1}H-NMR and ^{13}C-NMR shift values of compound **3**. ^{1}H- and ^{13}C- assignments were obtained from HSQC and HMBC experiments.

**Position**	**δ_H (ppm) (J Hz)**	**(HSQC) δ_C (ppm)**	**HMBC**
**Ar-H**	7.52 (m)	136.20	H: Ar
**Ar-H**	7.48 (m)	129.10	H: Ar
**PhCH-**	5.5 (s)	125.31	H: Ar
**C-1**	4.88 (d, J = 4.2)	105.09	H: 2, OCH_3
**C-2**	4.73 (dd, J = 3.7 and 9.7)	73.95	H: 1, 3
**C-3**	4.22 (t, J = 9.7)	74.25	H: 2, 4
**C-4**	3.51 (t, J = 9.7)	76.09	H: 3, 5
**C-5**	3.79 (ddd, J = 4.7, 9.7 and 14.1)	69.35	H: 4, 6a, 6b
**C-6a, 6b**	3.96 (dd, J = 4.7 and 10.1); 3.78 (t, J = 10.1)	62.05	H: 2, CO; H: 5, CO
**OCH_3**	3.42 (s)	58.06	H: 1
**$CH_3(CH_2)_9CH_2CO$-**	---	177.90	H: 1, 3

Table S2 Structures of synthesized methyl 4,6-*O*-benzylidene-α-D-glucopyranoside derivatives **2-7**.

Entry	Molecular formula	Structures
**2**	$C_{14}H_{18}O_6$	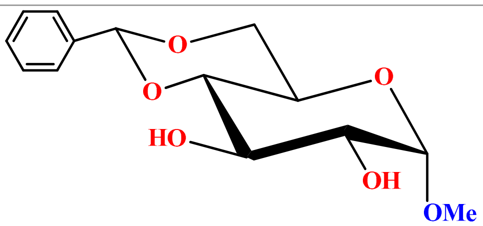
**3**	$C_{26}H_{40}O_7$	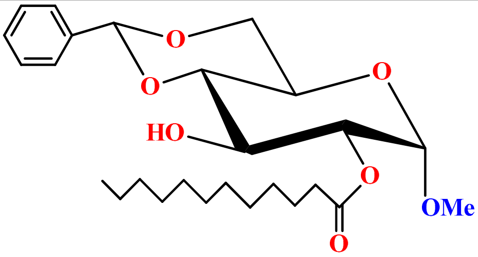
**4**	$C_{40}H_{66}O_8$	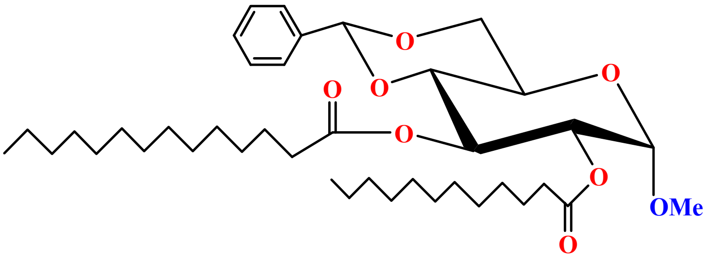
**5**	$C_{42}H_{70}O_8$	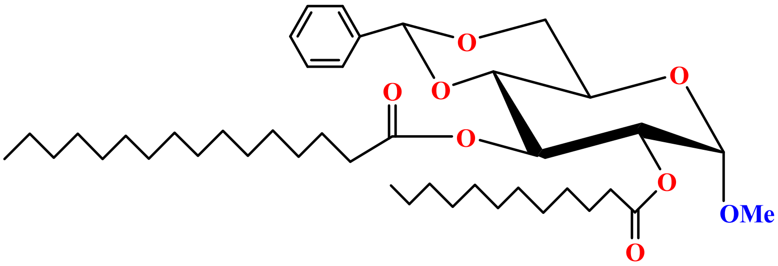
**6**	$C_{45}H_{54}O_7$	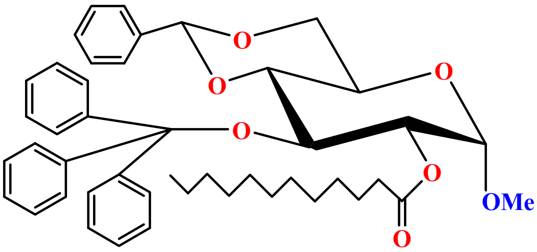
**7**	$C_{35}H_{46}O_8$	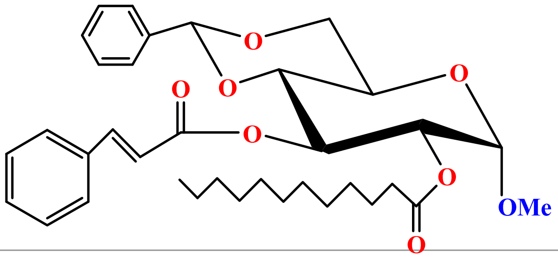

Table S3 MIC and MBC values in μg/ml compounds **4** and **7** against tested bacteria.

**MIC values in μg/mL**					
**Entry**	*P. aeruginosa*	*E. coli*	*S. abony*	*S. aureus*	*B. subtilis*
**4**	1350.00±0.01	1350.00±0.01	675.00±0.01	1350.00±0.01	675.00±0.01
**7**	1350.00±0.01	1350.00±0.01	675.00±0.01	1350.00±0.01	675.00±0.01
***Azithromycin**	225.00±0.01	225.00±0.01	240.00±0.01	215.00±0.01	250.00±0.01
**MBC values in μg/mL**					
**4**	2700.00±0.02	2700.00±0.02	5400.00±0.04	2700.00±0.02	2700.00±0.02
**7**	1350.00±0.01	5400.00±0.04	2700.00±0.02	2700.00±0.04	2700.00±0.02
***Azithromycin**	275.00±0.01	250.00±0.01	290.00±0.01	310.00±0.01	310.00±0.01

*Azithromycin = positive control

Table S4 Calculated frontier molecular orbital parameters of derivatives **2-7**.

Entry	HOMO	LUMO	ΔE	IP	EA	ἡ	μ	S	X	ω
2	-6.7840	-0.3665	6.4175	6.7840	0.3665	3.2087	-3.5753	0.1558	3.6784	0.9959
3	-6.9016	-0.4552	6.4463	6.9016	0.4552	3.2231	-3.6784	0.1551	3.9053	1.0494
4	-7.1258	-0.6849	6.4409	7.1258	0.6849	3.2204	-3.9053	0.1552	3.8755	1.1839
5	-7.0956	-0.6555	6.4401	7.0956	0.6555	3.2200	-3.8755	0.1552	3.4369	1.1661
6	-6.3114	-0.5624	5.7489	6.3114	0.5624	2.8744	-3.4369	0.1739	4.2732	1.0273
7	-6.5878	-1.9586	4.6292	6.5878	1.9586	2.3146	-4.2732	0.2160	4.2732	1.9723

Gap ΔE: LUMO-HOMO, IP (-HOMO): Ionization potential, EA (-LUMO): Electron affinity, X (*I*P+E*A*)/2: Electronegativity, η (IP−EA/)2: Chemical hardness, S (1/2ἡ): chemical softness, μ −(IP+EA)/2: Chemical potential, ω (μ2 /2η): Electrophilic index.

Table S5 iGEMDOCK protein-ligand interactions energy of compounds with fungal and bacterial target proteins.

Entry	Fungal target proteins		Bacterial target proteins	
	1KS5 (kcal/mol)	1R51 (kcal/mol)	4A1J (kcal/mol)	5IQR (kcal/mol)
2	-85.94	-84.51	-83.15	-103.03
3	-108.10	-106.05	-108.52	-104.54
4	-140.86	-116.11	-134.80	-121.84
5	-136.18	-117.41	-149.84	-130.56
6	-126.71	-112.70	-126.75	-122.84
7	-126.88	-118.50	-115.35	-120.40

Table S6 Calculated SwissADME parameters.

Parameters	2	3	4	5	6	7
MW	282.29	464.59	674.95	703.00	706.91	580.75
TPSA	77.38	83.45	89.52	89.52	72.45	72.45
RB	2	14	28	30	19	18
HBA	6	7	8	8	7	7
HBD	2	1	0	0	0	0
MLogP	-0.01	2.63	5.16	5.50	5.37	4.02
WLogP	-0.13	4.34	9.59	10.37	9.25	6.97
MR	67.49	125.30	192.72	202.34	203.37	164.45
GIA	High	High	Low	Low	Low	High
LipVi	0	0	2	2	2	1
GhoVi	0	1	4	4	4	4

MW: Molecular weight, TPSA: Topological Polar Surface Area, RB: Number of rotatable bonds, HBA: Number of hydrogen acceptors, HBD: Number of hydrogen donors, MLogP: Topological method, WLogP atomistic method octanol/water partition coefficient, MR: Molar refractivity, GIA: Gastrointestinal absorption, LipVi: Lipinski violations, GhoVi: Ghose violations.

Table S7 Strains of bacteria and fungi used for antimicrobial activity tests.

Types of organisms		Tested organisms and strains code	
Bacteria	Gram +ve	*Staphylococcus aureus*	ATCC 6538
		Bacillus subtilis	ATCC 6633
	Gram -ve	*Salmonella abony*	NCTC 6017
		Pseudomonas aeruginosa	ATCC 9027
		Escherichia coli	ATCC 8739
Fungi		*Aspergillus flavus*	ATCC 204304
		Aspergillus niger	ATCC 16404
